# COVID-19 and reproductive justice in Great Britain and the United States: ensuring access to abortion care during a global pandemic

**DOI:** 10.1093/jlb/lsaa027

**Published:** 2020-05-18

**Authors:** Elizabeth Chloe Romanis, Jordan A Parsons, Nathan Hodson

**Affiliations:** 1 Department of Law, The University of Manchester, UK; 2 Bristol Medical School, University of Bristol, UK; 3 T.H. Chan School of Public Health, Harvard University, USA

## Abstract

In this paper we consider the impact that the COVID-19 pandemic is having on access to abortion care in Great Britain (GB) (England, Wales, and Scotland) and the United States (US). The pandemic has exacerbated problems in access to abortion services because social distancing or lockdown measures, increasing caring responsibilities, and the need to self-isolate are making clinics much more difficult to access, and this is when clinics are able to stay open which many are not. In response we argue there is a need to facilitate telemedical early medical abortion in order to ensure access to essential healthcare for people in need of terminations. There are substantial legal barriers to the establishment of telemedical abortion services in parts of GB and parts of the US. We argue that during a pandemic any restriction on telemedicine for basic healthcare is an unjustifiable human rights violation and, in the US, is unconstitutional.

## I. INTRODUCTION

As governments worldwide continue to take extraordinary measures to address the COVID-19 pandemic, there has been a significant impact on access to reproductive health services. In this paper we consider how COVID-19 is impairing access to abortion care in Great Britain (GB) (England, Wales, and Scotland)^1^ and the United States (US). We demonstrate that there are substantial legal, geographical, social, and socioeconomic barriers for many people^2^ trying to access abortion care and that these access issues surrounding abortion care have become critical during the current emergency. We argue that, in order to ensure people can access essential healthcare, early medical abortion (EMA) services should be facilitated remotely. Telemedicine is being deployed in other routine health services, and it is appropriate, safe, and essential for this step to be made in abortion care. In parts of the US, however, restrictive regulation is preventing remote abortion care. This was also the case in GB until very recently. Changes to the law and health policy in the US are necessary to ensure access, and the recent changes in GB which we discuss require added permanence.

In this paper, we take it as given that abortion is essential healthcare in order to protect the physical and mental health of many people from the risks posed by unwanted pregnancy.^3^ Service provision is always necessary to meet the demand for abortion care, and demand will continue throughout the pandemic. Concerns about reproductive and sexual health are often neglected during emergencies. While we understand that governments are focused on managing the spread of COVID-19 and ensuring provisions to treat those infected, the issues we raise remain in need of attention. Access to services is a matter of reproductive justice^4^ that is, ‘the human right to maintain personal autonomy, have children, not have children and parent the children we have in safe and sustainable communities.’^5^ While reproductive justice is not concerned only with abortion access, and other aspects of reproductive justice will be affected by this pandemic, we have narrowed our scope to abortion.

Remote access to abortion services will be a global problem at this time. We narrow our focus to GB and the US, both common law jurisdictions, because there are or were specific legal barriers in operation here that we wish to highlight.^6^ The COVID-19 pandemic is having a huge impact on reproductive health, but law and policy in GB and the US is not shifting as it should to minimize the impact on people’s health.

## II. ACESSING ABORTION CARE IN A TIME OF COVID-19

Barriers to safe abortion access exist under normal circumstances, but they have been exacerbated by measures taken by governments to reduce the spread of COVID-19. Both GB and the US have geographical access inequalities in abortion care. In the US, the so-called abortion deserts necessitate travel of >100 miles to the nearest clinic, particularly in the South and Midwest.^7^ Such distances are considerably less common in GB, but there are parts of the country—especially in rural Scotland—with no local clinics. GB has also seen several clinic closures recently (prior and unrelated to COVID-19).^8^ Due to the pandemic, the number of potentially pregnant people living a significant distance from the nearest clinic has increased. The British Pregnancy Advisory Service (BPAS)—one of the independent abortion providers under National Health Service (NHS) contract which, combined, accounted for 72% of abortions in England and Wales in 2018^9^—has been forced to close 23% of their clinics due to virus-related staff shortages.^10^ In the US, states such as Ohio and Texas have deemed abortion non-essential,^11^ meaning people who would have attended a clinic in these states must now travel further.

Geographical inequalities have been worsened by policies of social distancing, as there are far more practical barriers to necessary travel. For people who already have children, arranging childcare is likely to be extremely challenging given school/kindergarten/nursery closures. Isolating relatives and caring responsibilities for older and vulnerable relatives may also increase. Making a long journey also heightens an individual’s risk of infection, particularly if it would entail the use of public transportation. This, in turn, puts those they live with and care for at a heightened risk. In-person provision also prevents those who work at abortion clinics from social distancing, putting them at risk of infection when a simple alternative is available.

BPAS estimated at the end of March 2020 that there were 44,000 people in GB who would need abortions during the period April to June 2020.^12^ This reflects the large number of lives affected by limiting abortion rights during the pandemic. Some of these people might make different decisions about their sex lives due to the pandemic, but we estimate that over 15,000 people in GB had already conceived by March 23, 2020, and would require an EMA during the lockdown.^13^ These 15,000 people conceived when abortion access was guaranteed by the government and are particularly vulnerable to the shifting changes in government policy on abortion care. They are at risk of either ending up continuing unwanted pregnancies or having second trimester abortions which have a higher rate of complications.^14^

The Centers for Disease Control and Prevention (CDC) carry out abortion surveillance in the US. The latest published statistics are from 2016 and incorporate data from 48 out of 52 reporting areas.^15^ 623,471 abortions were reported from these 48 reporting areas in 2016. 91% (567,358) were performed before 13 weeks.^16^ This means that delays during the 3 weeks between March 22 and April 12, 2020 could mean 32,732 abortions were affected or even delayed. If restrictions last 12 weeks that figure rises to 130,929. In Ohio alone there were 20,790 abortions in 2015;^17^ that amounts to 4,798 people affected over a predicted 12 weeks of restrictions.

We are not criticizing measures that governments continue to take in response to COVID-19, but noting that when such restrictions are placed on the movement of citizens, alternative provisions must be made to ensure essential services are provided. Abortion is an essential service, and governments have a duty to ensure access continues throughout this and any future pandemics.

## III. TELEMEDICAL ABORTION CARE

EMA involves the use of two medications to induce miscarriage and expulsion of the products of conception—mifepristone first, then misoprostol 24–48 hours later. There is, however, variation in gestational time limits set in different countries for accessing EMA.

Clinical guidelines developed by the National Institute for Health and Care Excellence (NICE) in England and Wales recommend EMA up to and including 10 weeks’ gestation.^18^ These guidelines note that before 9 weeks’ gestation, pregnant people might be offered the option of both medications together, although this has a higher risk of complications, which is reflected in the advice of BPAS.^19^ The UK Royal College of Obstetricians and Gynaecologists similarly recommends EMA until 9 weeks and 6 days,^20^ whereas the American College of Obstetricians and Gynaecologists recommends a lower limit of 9 weeks’ gestation.^21^ The World Health Organization (WHO) acknowledges the safety of EMA prior to 9 weeks and makes a ‘weak’ recommendation that EMA may be used up to 12 weeks’ gestation, noting that the quality of evidence for the latter is ‘low.’ ^22^ Although they differ regarding the exact cut-off, these organizations agree that EMA prior to 9 weeks’ gestation is appropriate. As such, to avoid confusion, any mention of EMA hereafter refers to EMA prior to 9 weeks’ gestation.

Telemedical provision of EMA (TEMA) is not a recent introduction, though remains uncommon. Where it is available, there exist several models of provision which vary greatly in the extent to which they improve access. In Iowa in 2008, a study enabled clinics to dispense drugs in person following remote authorization by an off-site physician.^23^ Started in 2016, the TelAbortion Project allows people in select states across the US^24^ to videoconference with an abortion provider before being posted EMA drugs.^25^ While these two approaches undoubtedly improve(d) access to abortion in the US, both required people to undergo tests and ultrasounds, thereby necessitating attendance at some form of healthcare facility.

Women on Web provide a fully remote service, utilizing an online question-based consultation method^26^ before posting the drugs to eligible persons.^27^ The service provided by Women on Web is for those in countries without safe or legal abortion services,^28^ but it could be adapted for TEMA during the COVID-19 pandemic. A fully remote option is best suited to abortion provision during the pandemic for the reasons already outlined, though we would suggest the inclusion of direct verbal contact between the user and physician either by phone or video call, not only to ensure understanding but to reassure people who may be anxious.

A recent systematic review assessed the ‘success rate, safety, and acceptability for women and providers of medical abortion using telemedicine.’^29^ In addition to finding it highly acceptable, service users experienced similar rates of complete abortion and adverse outcomes as with in-clinic provision. Further, the WHO recommends the use of checklists and pregnancy tests in self-assessment of abortion completeness,^30^ so an in-person follow-up consultation is not necessary unless there are complications.

## IV. LEGAL BARRIERS TO TELEMEDICAL ABORTION CARE

In this section, we outline the legal landscape regarding TEMA in both GB and the US. As the COVID-19 pandemic unfold, government responses to the crisis continue to develop. During the time of writing this paper, several changes have unfolded, only some of which are to be welcomed.

### A. Great Britain

In GB abortion remains a criminal offence^31^ and is lawful only where provided under the conditions detailed in the Abortion Act 1967 (AA 1967).^32^ Two doctors^33^ must, forming their opinion in good faith,^34^ believe that one of the grounds for termination in the Act is satisfied. The first of these is that the pregnancy has not exceeded 24 weeks and continuing the pregnancy would involve greater risk of injury to the woman’s^35^physical or mental health (and that of any existing children) than termination.^36^ EMA before 9–10 weeks’ gestation is very safe^37^ and much safer than the risks of unwanted pregnancy to a person’s physical and mental health.^38^ Until recently, the AA 1967 placed two barriers to remote provision of EMA. First, the requirement that ‘two’ doctors must certify a lawful abortion is far more difficult at present due to resources and safety of staff at clinics.^39^Second, the Secretary of State for Health and Social Care and health ministers of the devolved governments have the exclusive power to determine ‘where’ a lawful abortion may take place per sections 3 and 3A of the AA 1967.^40^ Approval orders were issued by the Scottish Minister for Public Health in 2017,^41^ Welsh Minister for Health in 2018,^42^ and UK Secretary of State for Health and Social Care in 2018,^43^ declaring that it is lawful for women to self-administer misoprostol at home but only if they have:

(i) Attended a clinic in order to be prescribed both mifepristone and misoprostol.
(ii) Been supervised administering mifepristone in the clinic.
(iii) Are ordinarily resident at the place where they self-administer misoprostol.^44^

These orders, therefore, expressly prevented people from accessing treatment remotely because attendance at a clinic is mandated for prescription and these orders meant it was unlawful for a woman to self-administer mifepristone at home (or anywhere other than a clinic or hospital).

On March 23, 2020, the UK Secretary of State for Health and Social Care issued a new approval order stating that, in England, a person could lawfully self-administer both mifepristone and misoprostol at home provided that they consult with a clinic or hospital or a registered medical practitioner via videolink, telephone conference, or other electronic means, and they are prescribed the medications to be taken for the purposes of terminating pregnancy.^45^ Only hours later, however, the order was revoked, removed from the government website, and replaced by a statement that the publication of the order was an ‘error.’^46^ The unlawfulness, however, of remote abortion care was at odds with government and healthcare regulators’ response to other aspects of routine healthcare where remote care were being encouraged.^47^ In response to an open letter by reproductive healthcare professionals,^48^ the Department of Health and Social Care again announced they were relaxing abortion regulations in England on March 30, 2020. Following the new approval order, pregnant persons can be prescribed both abortion medications by videolink, telephone, or any other electronic means and can take both medications in their homes where pregnancy has not 9 weeks and 6 days’ gestation.^49^ The new approval order is in force until March 30, 2022, or the day on which the temporary provisions in the Coronavirus Act 2020 expire (whichever comes first).^50^ On March 31, 2020, the Scottish^51^ and Welsh^52^ governments issued similar orders meaning that TEMA is now lawful throughout GB. It remains the case that two doctors’ signatures are necessary for service provision.^53^ Furthermore, we note that the delay in making this change has had a real impact on persons in GB who were seeking abortion at the time in both delaying treatment and causing severe distress.

The reason for the delay remains unclear. The significant difference between the two approval orders related to England appears only to be making specific provision for the period in which TEMA is lawful. The March 23 approval order specified no end date, whereas the March 30 order is explicit that the order expires without further government action. The approval order for Wales specifies that, like the English order, it expires 2 years after its issue date (March 31, 2022) or on the day on which the temporary provisions in the Coronavirus Act 2020 expire, ‘whichever is earlier.’^54^ The provision made for the automatic revocation of relaxed regulations surrounding TEMA thus means that regressive and *unnecessary* rules regarding the place of medical abortion will come back into effect without the government having to act. Notably, the approval order issued by the Scottish government does not have an expiry date; however, the Scottish Chief Medical Officer notes in an explanatory letter that ‘we intend that it will have effect for a limited period and so would revoke it and replace it with the terms of the previous approval (dated October 2017) at an appropriate time when it is judged that it is no longer necessary in relation to the pandemic response.’^55^ In Scotland, therefore, the order seems to have placed pregnant people’s health, rather than political expediency, at the center of their effort to ensure access for the foreseeable future, albeit without permanently committing to the change.

While we have limited our inquiry to those places in the United Kingdom (UK) where the AA 1967 applies (GB), we note here with concern that although *temporary* progressive changes have been made in all other parts of the UK, the situation remains very different in Northern Ireland. TEMA remains unlawful in Northern Ireland because the new framework for abortion, following the decriminalization of abortion in Northern Ireland in July 2019,^56^ came into force on March 31, 2020,^57^ and is specific that EMA is lawful only where prescribed and administered by a medical practitioner in general practitioners premises, health and social care (HSC) clinics, or hospital and women’s homes where the second medication of EMA may be taken.^58^ This provision explicitly prohibits TEMA by rendering the taking of mifepristone at home unlawful.^59^ Several abortion providers, however, have openly announced their intention to facilitate pregnant people’s access to TEMA despite the Northern Irish Department of Health’s failure to legalize^60^ on the grounds that it is necessary to prevent grave, permanent injury to the physical or mental health of pregnant persons as permitted under regulation 11 of the new regulations.

### B. The United States

Ironically, given the fact that access to abortion is often far more difficult for people in parts of the US, Americans, unlike British people, have a constitutional right to abortion access. In *Roe v Wade*,^61^ the US Supreme Court affirmed that the Constitutional right to privacy encompassed a right to terminate a pregnancy (though qualified at the point of viability by the state’s interest in potential life).^62^ In *Planned Parenthood v Casey*,^63^ the Supreme Court reaffirmed a person’s right to access abortion as part of the right to privacy, though replacing the ‘trimester framework’ adopted by the Court in Roe with the ‘undue burden test.’ They held that a law is incompatible with the constitution if its ‘purpose or effect is to place a substantial obstacles in the path of a woman seeking an abortion before the fetus attains viability.’^64^ Individual states are free to pass whatever laws they see fit regulating abortion, provided that those laws do not *unduly* prevent access to abortion before the viability threshold.^65^ Abortion is *legal* in every state, but there are different restrictions on provision.

Several states have expressly banned TEMA services: Arizona,^66^ Arkansas,^67^ Indiana,^68^ South Carolina,^69^ and West Virginia.^70^ Other states have laws that, while making no specific reference to telemedicine, render TEMA unlawful because of specific requirements (usually rules mandating the physical presence of the physician during abortion): Alabama,^71^ Oklahoma,^72^ Louisiana,^73^ Mississippi,^74^ Missouri,^75^ Nebraska,^76^ North Carolina,^77^ North Dakota,^78^ South Dakota,^79^ Tennessee,^80^ Texas,^81^ and Wisconsin.^82^ These states have adopted an approach very similar to that taken in GB before the recent changes outlined. The Supreme Court of Iowa, in reference to law in force in Iowa at the time requiring a physician to perform a physical examination and be physically present when abortion-inducing drugs are provided, noted that ‘it is not disputed that this rule would have the effect of prohibiting telemedicine abortions.’^83^

Since the beginning of the COVID-19 emergency, further action has been taken by state legislatures that have placed further restrictions on access to abortion care at this time. In Texas, where TEMA is already unlawful,^84^ the governor issued an executive order stating that ‘all licensed healthcare professionals and all licensed healthcare facilities shall postpone all surgeries and procedures that are not immediately necessary to correct a serious medical condition of, or to preserve the life of, a patient who without immediate performance of the surgery or procedure would be at risk for serious adverse medical consequences or death, as determined by the patient’s physician.’^85^ The Texas Attorney General confirmed that abortion procedures not medically necessary to preserve the life or health of the mother must be postponed.^86^ The governor has since made some exceptions to this order allowing abortion clinics to resume providing care.^87^ Mississippi, where TEMA is also unlawful,^88^ has also taken these same additional steps.^89^ The Ohio Senate passed a bill banning telemedical abortion on March 4 this year,^90^ despite the emerging evidence that remote healthcare was becoming increasingly important. A similar executive order requiring the postponement of nonessential medical services was also issued,^91^ and the Deputy Attorney General wrote to clinics ordering their closure as ‘a necessary measure amid a public health crisis.’^92^ State legislatures have been accused of ‘exploiting the COVID-19 crisis to further their agenda’ to prevent access to abortion care.^93^ The attempts to secure closure of clinics makes introducing TEMA provision all the more pressing.

## V. SECURING JUSTICE: THE CASE FOR TELEMEDICAL ABORTION CARE DURING COVID-19

TEMA is necessary during the pandemic to ensure access to treatment, as a matter of safety and a matter of justice.

### A. Ensuring Access to Treatment

While TEMA is a positive, evidence-based addition to reproductive healthcare generally, during the COVID-19 pandemic it is a necessary means of ensuring continued access to an essential treatment. As earlier outlined, it is extremely difficult for some people to access clinics during social distancing measures. TEMA is a simple means of providing abortion services for the many people estimated to seek a termination of pregnancy in the coming months,^94^ as well as removing the potential stress of trying to reach a clinic. In the US, TEMA is currently available in only 12 states. ^95^ While not specifically speaking on the matter of abortion, Justice Brandeis commented in *New State Ice Co. v. Liebmann* that a state could, ‘if its citizens choose, serve as a laboratory; and try novel social and economic experiments’.^96^ Although there is no constitutional requirement for consistency in abortion care provision between the states, policy debates across the US should take into account best practice emerging from ‘experiments’ such as TEMA.

In GB, the recent orders to allow TEMA mean that organizations such as BPAS are now providing EMA medications by post following a telephone consultation and medical assessment.^97^ The lack of existing operational infrastructure, in addition to the increased operational complications of the current pandemic, means that the new system is likely to see challenges, though such providers have long been calling for the introduction of TEMA and are fully committed. Regardless, the challenges of establishing a new system during a pandemic are *not* grounds enough to withhold TEMA. That the TelAbortion Project is already in operation in the US suggests that implementing in additional states would, from a practical, operational perspective, be simpler than it was in GB.^98^

### B. A Matter of Safety

TEMA protects people seeking abortion care by ensuring prompt access during the pandemic. Even if individuals are still able to access abortion services in-person during the pandemic, but later than they otherwise would have, the potential risks associated with that abortion are greater given the time sensitivity of EMA earlier outlined.

Among those who are entirely unable to attend an abortion clinic due to social distancing, caring responsibilities, clinic closures, and reduced transport system operations, the majority are likely to eventually require a surgical abortion. Although later extended, restrictive measures in GB were initially implemented for a period of 3 weeks, and those in the US are likely to persist at least as long.^99^ Given the gestational restrictions on EMA any person who is currently 6 weeks pregnant would no longer have the option of a medical abortion after the 3 weeks of social distancing—and that is assuming measures are not extended. Where social distancing means that a pregnant person crosses the threshold of 9 weeks, they *may* still be able to have an EMA but are unnecessarily pushed into a more high-risk group.^100^

Objections to TEMA based on safety concerns are unfounded under normal circumstances, but during current pandemic measures they are even less compelling.^101^ The balance of benefits and harms tips even more clearly in favor of TEMA’s introduction when leaving the house to attend a medical clinic puts oneself and one’s family at risk of COVID-19 infection. TEMA is a safe, effective, and acceptable option which not only protects the safety of people in reducing the likelihood of a need for more invasive and less safe abortions, but also removes the need for people to break from social distancing and risk becoming infected with, or exposing others to, COVID-19.

### C. A Matter of Justice

Those who are most impacted by the barriers we have outlined—geographical, physical, and social—are those who we might consider extremely vulnerable. This involves groups of people who might be structurally disadvantaged as a result of their gender, socioeconomic status, disability, chronic illness, or significant caring responsibilities. There is a long-standing concern about the ability of such individuals to access abortion services, and the extent to which those in the US may rely on the ‘undue burden’^102^ standard is questioned, particularly in light of the so-called TRAP (targeted regulation of abortion providers) laws.^103^ Remote access will be the only way, during this crisis and beyond,^104^ to ensure that vulnerable minorities are able to access care.

That the orders permitting TEMA in GB have sunset clauses^105^ is problematic in terms of securing lasting reproductive justice. It further demonstrates that restrictions on TEMA were not evidence-based, as if there were serious medical concerns it would not have been permitted in response to the pandemic. The introduction of TEMA as a response to COVID-19 suggests that it was simply to prevent those seeking a termination from breaking social distancing measures, meaning that the previous requirement of clinic attendance was purely become people *could* attend. For changes which improve access for so many people in GB to be time limited is, then, a fleeting victory. Only with an alteration that incorporates permanence will the pandemic-induced relaxation of regulation in GB ensures protection of disadvantaged individuals seeking abortion services. The temporary changes, then, are a step in the right direction which, it is hoped, will prove to be a catalyst for lasting progress.

## VI. CONSTUTIONALITY OF RESTRICTIONS ON TELEMEDICAL EARLY MEDICAL ABORTION

In this section, we consider how restrictions on TEMA in GB were (and restrictions in Northern Ireland remain) a potential violation of the human right to private life and to freedom from degrading treatment, and how the US restrictions violate the right to privacy contained in the US Constitution.

### A. Great Britain

The vast majority of jurisprudence considering the compatibility of the law concerning abortion in GB and Northern Ireland with the European Convention on Human Rights (ECHR) concerns the right to private life. This right, contained in Article 8 of the ECHR and the Human Rights Act 1998, ‘cannot be interpreted as conferring a right to abortion’^106^ according to the European Court of Human Rights (ECtHR). Though, the court has found that signatory states must have an effective and accessible procedure for people to receive care where pregnancy threatens life.^107^ There has also been a clear evolution in ECHR jurisprudence^108^ from the insistence that interference with decisions about pregnancy termination does not necessary constitute an interference with the right to private life^109^ to the belief that interference with abortion provision does engage Article 8.^110^ There are both, as Scott notes, negative and positive obligations of the state arising under Article 8 in the context of abortion: negative obligation being ‘non-interference with’ and positive obligations being ‘respect for’ private life.^111^

In this paper, we are not making an argument about whether people have the right to abortion^112^ but rather that the government unnecessarily delaying access to lawful treatment, ultimately meaning that a person has to incur more risk in that treatment, or potentially entirely denying access to lawful treatment altogether, is a substantive interference with the right to private life.^113^ Thus, we are concerned with the positive obligations of the state in relation to TEMA during COVID-19: asking whether legal arrangements for care are sufficient to protect a person’s interest in physical and psychological integrity.^114^ The ECtHR has held that ‘once a legislature decides to allow abortion, it must not structure its legal framework in a way which would limit real possibilities to obtain it.’^115^ The ‘key legal point,’ Scott stresses, ‘as regards the UK’s positive obligations, is that once a state has said that abortion is lawful on certain grounds, it must protect or guarantee the availability of abortion on those grounds, since its margin of appreciation [as recognised by the ECtHR] in relation to abortion will have been significantly reduced.’^116^ In *RR v Poland*,^117^ a case concerning access to prenatal screening for the purposes of assisting in an abortion decision, this point was pivotal; the ECtHR’s decision was clear that because Polish law did allow for abortion in cases of fetal malformation ‘there must be an adequate legal and procedural framework to allow the relevant, full and reliable information on the foetus’s health be made available to the pregnant woman.’^118^ This ruling effectively stipulates that signatory states are under a positive obligation to organize health services in such a way that ensures that individuals are able to access the abortion services to which they are legally entitled.

Before the UK government, and devolved governments’, changes to the law on March 30 and 31, 2020, there was clearly a substantive failure by the state to meet their obligations under Article 8 of the ECHR. Provision of EMA, where two doctors agree,^119^ clearly satisfies the first criterion for lawful abortion provision as the risks associated with EMA within the time scale advocated are far fewer than those associated with pregnancy and childbirth.^120^ The argument regarding the state’s positive obligations related to abortion we have here advanced, we believe, stands even once lockdown has been relaxed, and the provisions of the Coronavirus Act 2020 cease to have effect. It is likely to be the case that social distancing may limit individuals’ abilities to access clinics for some undefined time into the future and social and geographical barriers to care that existed in GB prior to the pandemic also mean that the organization of health services in the prevention of TEMA is a failure to adhere to human rights standards. It is also the case that pregnant people in Northern Ireland continue to have their right to private life denigrated by the state.

The failure to facilitate privacy rights that was evident in March 2020 in GB was unlikely to have been justified by one of the derogations contained in Article 8 (2) of the ECHR.^121^ The unlawfulness of TEMA certainly cannot be justified on the grounds that it is necessary for the protection of health, especially in the COVID-19 context as remote care is clearly better for the health of a person needing an abortion themselves, their families, staff at abortion clinics, and others. The UK is entitled, under Article 15 of the ECHR, in times of ‘exceptional... crisis or emergency which affects the whole population and constitutes a threat to the organised life of the community of which the State is composed,’^122^ to derogate from their obligation to secure rights to private life^123^ including positive obligations. However, states are only able to do so if they comply with procedural requirements to invoke Article 15^124^ (which the UK has not yet) and that such a derogation is required by the emergency at hand.^125^ We have submitted that it is in fact a necessity of the situation that people be afforded access to TEMA.

We also believe that there may also be a compelling case that the denial of TEMA care for the duration of March 2020 (and that continues in Northern Ireland) might constitute inhuman or degrading treatment under Article 3 of the ECHR. Inhuman treatment is potentially premeditated treatment of long duration, causing serious physical and mental suffering or acute psychiatric distress.^126^ Degrading treatment incites feelings of fear, anguish, and inferiority, which is humiliating and breaks physical or moral resistance.^127^ There is a substantial body of case law establishing that the state owes a responsibility to provide appropriate healthcare to prisoners because of their lack of liberty and total reliance on the state for access to care.^128^ Given the circumstances and restrictions on individual liberty currently in place in GB (and were at the time that TEMA remained unlawful), and parts of the US, the substantial impact on liberty is in some ways similar; being effectively denied healthcare during social distancing and isolation measures is somewhat comparable. Importantly, the state is not being asked to facilitate this care directly, merely to remove restrictions that prevent abortion providers from providing basic care.

In order to demonstrate that denial of medical care amounts to inhuman or degrading treatment a minimum threshold of severity must be met.^129^ The ECtHR holds that severity ‘depends on all the circumstances of the case, such as the nature and context of the treatment, the manner of its execution, its duration, its physical or mental effects, and in some instances, the sex, age and state of health of the victim.’^130^ In the context of abortion, the ECtHR has thus far been largely unwilling to accept arguments pertaining to inhuman and degrading treatment because they were not thought to meet the threshold of severity necessary to demonstrate treatment is bad enough. In *A, B and C v Ireland*^131^, the applicants argued that ‘overcoming taboos to seek an abortion abroad and aftercare at home or maintaining the pregnancy in their situations—were degrading and a deliberate affront to their dignity’^132^ and that the Irish government was under a positive obligation to protect them from this. The court noted that travelling abroad to access abortion was ‘psychologically and physically arduous’ for all in such a situation and may even been financially burdensome for some.^133^ However, they reiterated the threshold of severity and concluded only that ‘the facts alleged do not disclose a level of severity failing within the scope of Article 3.’^134^ Notably the ECtHR did not provide substantive reasons as to why. In *Tysiąc v Poland* the claimant argued that her Article 3 rights were violated by ‘the failure of the state to make a legal abortion possible in circumstances which threatened her health, and to put in place the procedural mechanism necessary to have her right realised, meant that the applicant was forced to continue with a pregnancy for 6 months knowing that she would be nearly blind by the time she gave birth.’^135^ While the court found that her anguish and distress ‘could not be overstated’^136^, they found no breach of Article 3 considering instead that the complaints were ‘more appropriately examined under Article 8.’^137^ However, again no substantive reasons were provided as to why the treatment of the claimant did not meet the threshold of severity in Article 3.

There is one notable exception to the ECtHR’s pattern of denying that Article 3 is engaged without substantive consideration of the reasons why. In *RR v Poland*, thorough examination was given to the claimant’s case that her Article 3 rights were violated where she was denied access to abortion.^138^ The claimant argued that her Article 3 rights were engaged by an intentional failure by medical professionals to provide necessary medical treatment, here being timely prenatal examination that would have allowed her to find out necessary information about any fetal abnormality in time to make a decision about abortion within the time limits dictated by Polish law. In this case the ECtHR conducted a thorough examination of the circumstances of the case and determined that the claimant had in fact been subjected to degrading treatment, because of her situation being one of ‘great vulnerability,’^139^ resulting in ‘weeks of painful uncertainty,’^140^ and ‘acute anguish through having to think about how she and her family would be able to ensure the child’s welfare.’^141^ They noted in particular that she was ‘so shabbily treated by doctors dealing with her case’^142^ and that ‘no regard was had for the temporal aspect of the applicant’s predicament.’^143^ This was found to be particularly concerning because the Polish government did not attempt to argue that ‘at the material time genetic testing as such was unavailable for lack of equipment, medical expertise or funding.’^144^

The legal barriers on TEMA that remained in place for some time after lockdown was instigated in GB (and that remain in Northern Ireland), despite there being a clear safety and justice need to provide care during this time and onwards were, we submit, inhuman treatment for the purposes of the ECHR. The denial of treatment will continue to impact pregnant people for a long time, and the physical symptoms of early pregnancy^145^ combined with the mental health impacts of unwanted pregnancy,^146^ and stress in trying to obtain an abortion,^147^ will cause many people substantial suffering. These factors, we believe, can be likened to those experienced in *RR*. A failure to allow TEMA during a pandemic causes a significant delay to care without due regard to the time sensitivity involved in the provision of EMA. Moreover, it is notable that as a decision on the temporary lawfulness of TEMA was delayed, providers were both readying and willing to provide it; thus the inaction by the UK and devolved governments can be likened to the inaction of the doctors in *RR*. During the current emergency, pregnant and potentially pregnant people are experiencing real uncertainty in a multitude of ways in various aspects of their lives. Widespread economic uncertainty is a particular concern for many, who worry that it may affect their livelihood. This may be related to, or exacerbate stress experienced in relation to, concerns about the ability to afford or care for a child during the current conditions or in the post-COVID-19 world. Concerns that a pregnant person might have about a potential future child’s welfare and the care they can provide were a pertinent part of the decision in *RR*. Some of these factors we have outlined might remain pertinent after lockdown restrictions and social distancing restrictions are lifted, and in England and Wales, TEMA automatically becomes unlawful once more.

Although TEMA is now temporarily lawful in England and Wales, and lawful in Scotland (though with an indication that it is temporary), it is still notable that the prohibition of TEMA that remained after lockdown measures were introduced in March 2020 is *still* causing people in need of access to care substantial fear and feelings of degradation. This is because there are still some potential service users who are unsure of whether they are able to access abortion lawfully because of the government U-turns on this issue and misreporting about the legalities of TEMA in mainstream media.^148^ Article 15 does not allow states to derogate from the prohibition of torture, inhuman, and degrading treatment.^149^

### B. United States

As noted, the ratio from the US Supreme Court in *Casey* stipulates explicitly that ‘[u]nnecessary health regulations that have the purpose or effect of presenting a substantial obstacle to a woman seeking an abortion impose an undue burden on the right.’^150^ The Supreme Court has been willing to accept as constitutional several restrictions passed on abortion access, including those that specifically have the effect of delaying access to treatment.^151^ In 2016, in *Whole Women’s Health v Hellerstedt*,^152^ the court rearticulated the ‘undue burden’ standard and was explicit that the standard requires a two-stage test to determine the constitutionality of a state legislative provision restricting abortion. First, there must be consideration of whether the law has the effect of placing an undue burden on pregnant people in accessing abortion when considered against the potential benefits.^153^ Second, the legitimacy of the interest a state is asserting in creating the law must be considered.^154^ This second point—the consideration of the state’s motivations for acting ‘in addition to’ whether there is an undue burden for access—constitutes significant movement on the articulation of the ‘undue burden’ standard. On this, Fetrow notes that there are limited interests that the state can assert in placing burdens on access before viability—health of the pregnant person, integrity of the medical profession, and the promotion of fetal life.^155^ We consider in what follows whether TEMA bans constitute an undue burden on access, and the legitimacy of state motivations in banning TEMA.

When applying the first limb of this test, the Supreme Court has emphasized, in both *Gonzales*^156^ and *Hellerstedt*, the weight that must be afforded to medical evidence^157^ in considering the potential benefits and burdens of legislation placing obstacles to access. TEMA bans (or requirements for physical examination) have been upheld by State Courts of Appeal, in Arizona on the grounds that in-person care was more effective,^158^ and in Arkansas on the grounds that the ban was not an ‘undue burden’ for a significant number of people.^159^ We submit that neither of these grounds justifying such bans can apply in the current emergency, as we have demonstrated that in-person care is unlikely to be as safe for patients^160^ or providers and that these restrictions are likely to prevent a significant number of people from accessing care or from accessing timely and safe care. It is evident that legal measures banning or limiting TEMA are having an unprecedented impact on access—far greater than those of concern to the courts in these two cases—during this pandemic and thus we believe they are unconstitutional by virtue of the test stipulated by the US Supreme Court. In *Hellerstedt*, the US Supreme Court found that other requirements in Texas abortion law, requiring doctors providing abortions to have admitting privileges to local hospitals and for clinics to be surgical centers, constituted an undue burden and thus were unconstitutional. The justices based their decision on the fact that the measures resulted in a significant drop in the availability of services (specifically citing fewer resources and increased crowding).^161^ The Supreme Court focused on the fact that there was limited evidence that doctors needed admitting privileges because the risk of complications in abortion care is low.^162^ This also applies in the context of TEMA bans—the medical evidence overwhelmingly supports TEMA, especially amid a pandemic. Furthermore, the concerns about crowding in clinics, limited clinics, and travelling distances are also even more applicable, and thus, during the pandemic (and potentially beyond), TEMA bans should be understood as an undue burden on access.

Second, we must consider the motivations of states in banning TEMA. *Roe v Wade*^163^ explicitly allows states to require that a doctor be involved in supervising or performing an abortion. The motivation behind TEMA bans is purported by states to be the preservation of pregnant people’s health, because it is argued that in-person care is safer.^164^ This argument has not been tested in the Supreme Court specifically on the issue of TEMA; however in *Hellerstedt*, in relation to Texas’s attempt to require that all abortion clinics be surgical centers, it was held that the state cannot require that an abortion take place in a particular place if it cannot be established that so doing would be of benefit to pregnant people’s health.^165^ We find it hard to understand how it could be articulated that a requirement that a pregnant person attend a clinic, as opposed to accessing care remotely, can be better for their health in the midst of a pandemic. The Iowa Supreme Court found that legislative intervention into TEMA services was unconstitutional because there was a lack of medical support for the measure.^166^ Justice Wiggins emphasized that the undue burden test is context-specific.^167^ We have demonstrated throughout this paper that there is overwhelming evidence that TEMA is safe and effective and the involvement of a doctor in person is not necessary to ensure this safe and effective care. Insofar as the involvement of a doctor is deemed necessary,^168^ remote consultation is sufficient to ensure appropriate medical supervision proportionate to the aim of ensuring people’s health without causing an undue burden on access.

In Texas, the Center for Reproductive Rights (CRR) and others recently (March 24, 2020) filed a lawsuit challenging the Texas Attorney General’s interpretation of the executive order that abortion is not essential healthcare on the grounds that it is unconstitutional. In the District Court on March 30, 2020, the state’s attempt to close abortion clinics was blocked by Judge Yeakel on the grounds that it amounted to an unconstitutional pre-viability abortion ban. This decision, however, was overturned by the fifth circuit with Judge Duncan concluding that ‘*all* constitutional rights may be reasonably restricted to combat a public health emergency.’^169^ Elisabeth Smith at the CRR maintains that ‘there is no pandemic exception to constitutional rights’ including that of access to abortion before viability.^170^ On April 11, 2020, Planned Parenthood, the CRR, and others filed an emergency application to the Supreme Court on the matter.^171^ This led many to speculate that this suit might be the opportunity the newly conservative Supreme Court had been seeking to review *Roe v Wade*.^172^ Since this paper was first submitted, the Texas governor has relaxed the restrictions that were an attempt to close abortion clinics, allowing them to remain open provided that they do not request any personal protective equipment be provided by public authorities.^173^

In April 2020, the Supreme Court overturned a long-standing precedent declaring that state juries must be unanimous in order to convict a criminal defendant.^174^ In his concurring opinion, Justice Kavanaugh advances his account of precedent binding the Supreme Court in which he stresses that ‘all justices now on the Court agree that it is sometimes appropriate for the Court to overrule erroneous decisions… some of the Court’s most notable and consequential decisions have entailed overruling precedent.’ In making this remark he explicitly references Roe v Wade and Casey in a footnote among other significant precedents relating to liberal rights.^175^ We have provided a persuasive case that the unavailability of TEMA during COVID-19 is unconstitutional; however we acknowledge that with the political realities of the present Supreme Court that the recognition of abortion rights that we are advocating for may be difficult to secure.^176^

## VII. CONCLUSION

The COVID-19 pandemic is having an unprecedented impact on access to healthcare. There are currently substantial geographical, physical, and legal barriers that are making it increasingly difficult for people in need of abortion care to access essential services. This is a direct result of social distancing measures, a reduction in the availability of transport to clinics, increasing caring responsibilities for family and children, and the need, in some cases, to self-isolate. In GB, the law that had initially prevented healthcare providers from establishing TEMA services during the current emergency has now been relaxed. There are a significant number of states in the US that have either directly banned TEMA or have passed law, much like GB (temporary measures aside), which prohibits it in practice. In this paper, we argued that the restrictions in GB before the temporary change in the law were a violation of the UK government’s positive obligation to respect the right to private life and potentially a failure to ensure freedom from degrading treatment, contained in the ECHR. The failure of some states in the US to permit TEMA is, we argue, a violation of the constitutional right to privacy (interpreted as encompassing a right to abortion access).

The present emergency circumstances do mean that governments are required to take extraordinary measures to protect public health; however interference with individuals’ access to essential and basic care is unjustified if it is not necessary in the circumstances. TEMA is necessary during COVID-19 to address the real access barriers to care that are increasingly evident. Moreover, allowing service providers to continue these services does no damage to the effort to curtail this pandemic; in fact, ensuring these services are available remotely is safer to prevent people travelling—exposing themselves and others to infection risk—to access care, and to prevent healthcare workers being unnecessarily exposed.

The barriers to care we have outlined are a matter of reproductive justice. While removing the legal barriers to TEMA does not automatically ensure access to care is guaranteed for lots of vulnerable people, it is a step in the right direction that we implore legislatures to address immediately.

## Footnotes

1 We do not refer to Northern Ireland because the Abortion Act 1967, which creates the restrictions that limit access to abortion care in Great Britain, does not apply to Northern Ireland. It should be noted that new regulations that have just come into force in Northern Ireland that also preclude telemedical early medical abortion.

2 The issues we raise are relevant to all persons with female biology who have the physiology to get pregnant irrespective of the gender they identify with. We recognize that the term ‘woman’ is both too narrow to encompass all those persons who worry about unwanted conception and too broad in that it encompasses many people who do not have the physiology to become pregnant. Where we have used the term ‘woman,’ it is because that language is specifically deployed in the law.

3 Tamara Hervey & Sally Sheldon, *Abortion by Telemedicine in the European Union*, 145 Int. J. Gynecol. Obstet. 125, 126 (2019).

4 L. Ross & R. Solinger, Reproductive Justice 9, (University of California Press, 2017).

5 SisterSong, *Reproductive Justice*, https://www.sistersong.net/reproductive-justice (accessed Mar. 22, 2020).

6 Remote access is an issue concerning reproductive services in other countries, such as Canada and New Zealand. However, since in these countries abortion is not criminally regulated, there are no legal barriers to the provision of services that exacerbate access concerns. We note that our observations about access issues being exacerbated by the pandemic and argument that remote services should be provided obviously apply, but we need not make observations about the law in these jurisdictions.

7 Alice Cartwright et al., *Identifying National Availability of Abortion Care and Distance From Major US Cities: Systematic Online Search*, 20 J. Med. Internet Res. e186.

8 Charlotte Paxton, *Fears Women will ‘Have to Travel Further for Care’ as Five Abortion Clinics in Birmingham and the Black Country Shut*, https://www.birminghammail.co.uk/news/midlands-news/fears-women-have-travel-further-17535056 (accessed Mar. 20, 2020).

9 Department of Health and Social Care, *Abortion Statistics England and Wales: 2018*, https://assets.publishing.service.gov.uk/government/uploads/system/uploads/attachment_data/file/808556/Abortion_Statistics__England_and_Wales_2018__1_.pdf (accessed Mar. 23, 2020).

10 British Pregnancy Advisory Service, *Healthcare Professionals Call on Boris Johnson to Intervene to Protect Women’s Health—Reckless Failure to Listen to Scientific Advice is Putting Vulnerable Women at Severe Risk*, https://www.bpas.org/about-our-charity/press-office/press-releases/healthcare-professionals-call-on-boris-johnson-to-intervene-to-protect-women-s-health-reckless-failure-to-listen-to-scientific-advice-is-putting-vulnerable-women-at-severe-risk/ (accessed Mar. 25, 2020).

11 Texas Attorney General, *Healthcare Professionals and Facilities, Including Abortion Providers, Must Immediately Stop All Medically Unnecessary Surgeries and Procedures to Preserve Resources to Fight COVID-19 Pandemic*, https://www.texasattorneygeneral.gov/news/releases/health-care-professionals-and-facilities-including-abortion-providers-must-immediately-stop-all#.XnkW2CKrfO8.twitter (accessed Mar. 26, 2020); Ohio Department of Health, *Director’s Order for the Management of Non-essential Surgeries and Procedures Throughout Ohio*, https://content.govdelivery.com/attachments/OHOOD/2020/03/17/file_attachments/1403950/Director%27s%20Order%20non-essential%20surgery%203-17-2020.pdf (accessed Mar. 26, 2020).

12 British Pregnancy Advisory Service, *supra* note 10.

13 This figure is based on 205, 295 early medical abortions in the England and Wales in 2018 obtained from the Department of Health and Social Care, *supra* note 9 and ISD Scotland, ‘Termination of Pregnancy Year ending 2018’. https://www.isdscotland.org/Health-Topics/Sexual-Health/Publications/2019-05-28/2019-05-28-Terminations-2018-Report.pdf (accessed Mar. 29, 2020). The calculation of this figure assumes even distribution of abortions over the first 10 weeks’ gestation. This may not be true since many people do not discover their pregnancy until 5 weeks’ gestation, so this is likely to be an underestimate.

14 Karima Sajadi-Ernazarova and Christopher Martinez, *Abortion Complications*, https://www.ncbi.nlm.nih.gov/books/NBK430793/ (accessed Mar. 26, 2020).

15 The reporting areas include the 50 states plus the District of Columbia and New York City. The 48 reporting areas included in these statistics exclude California, the District of Columbia, Maryland, and New Hampshire.

16 Tara Jatlaoui et al., *Abortion Surveillance—United States*, 68 Surveill. Summ. 1 (2016).

17
*Id.*

18 National Institute of Health and Care Excellence, *Abortion Care*, https://www.nice.org.uk/guidance/ng140 (accessed Apr. 28, 2020) ¶ 1.9.

19 British Pregnancy Advisory Service, *Medical Abortion: The Abortion Pill Up to 10 weeks*, https://www.bpas.org/abortion-care/abortion-treatments/the-abortion-pill/abortion-pill-up-to-10-weeks/ (accessed Apr. 28, 2020).

20 Royal College of Obstetricians and Gynaecologists and British Society of Abortion Care Providers, *Clinical Guidelines for Early Medical Abortion at Home—England*, https://www.rcog.org.uk/globalassets/documents/guidelines/early-medical-abortion-at-home-guideline-england.pdf (accessed Apr. 28, 2020) at 4.

21 American College of Obstetricians and Gynaecologists, *Medical Management of First-Trimester Abortion*, https://www.acog.org/clinical/clinical-guidance/practice-bulletin/articles/2014/03/medical-management-of-first-trimester-abortion (accessed Apr. 28, 2020).

22 World Health Organization, *Safe abortion: Technical and Policy Guidance for Health Systems*, https://apps.who.int/iris/bitstream/handle/10665/70914/9789241548434_eng.pdf?sequence=1 (accessed Mar. 23, 2020) at 3.

23 Daniel Grossman et al., *Effectiveness and Acceptability of Medical Abortion Provided Through Telemedicine*, 118 Obstet. Gynecol. 296 (2011).

24 At the time of writing, the TelAbortion project operates in Colorado, Georgia, Hawaii, Illinois, Iowa, Maine, Minnesota, Montana, New Mexico, New York, Oregon, and Washington.

25 Elizabeth Raymond et al., *TelAbortion: evaluation of a direct to patient telemedicine abortion service in the United States*, 100 Contraception 173 (2019).

26 Service users are asked about their menstrual history to date their pregnancy, which is something it has been demonstrated that people are able to do reliably; see Roopan Gill & Wendy Norman, *Telemedicine and Medical Abortion: Dispelling Safety Myths, with Facts*, 4 Mhealth (2018).

27 Women on Web, https://www.womenonweb.org/ (accessed Mar. 30, 2020).

28 Women on Web is often noted as providing its services only to those in countries where abortion is illegal, though this is not strictly true. On their website (*Id.*), they claim to help those who ‘live in a country where access to safe abortion is restricted’ but not necessarily illegal. Indeed, the service is still available to individuals in Northern Ireland despite the recent legalization of abortion in the country.

29 M. Endler et al., *Telemedicine for Medical Abortion: A Systematic Review*, 126 BJOG 1094 (2019) at 1095.

30 World Health Organization, *Health Worker Roles in Providing Safe Abortion Care and Post-Abortion Contraception*, https://apps.who.int/iris/bitstream/handle/10665/181041/9789241549264_eng.pdf?sequence=1&fbclid=IwAR2Cm4YA4JEvu5asiVnT33kbXX7plXOXk1iTikfUlgyl8Uy2NWGuKuU6FY4 (accessed Mar. 30, 2020) at 41.

31 SS.58 and 59 Offences Against the Person Act 1861. We note that the Offences Against the Person Act 1861 does not apply in Scotland, however procuring miscarriage remains a criminal offence in Scotland by virtue of the common law.

32 As amended by S.37 Human Fertilisation and Embryology Act 1990.

33 S.1 (1) Abortion Act 1967, as amended by s.37 Human Fertilisation and Embryology Act 1990.

34
*Id.* ‘Good faith’ is interpreted broadly: see *R v. Smith (John Anthony James)* 58 Cr App r 106 and *Paton v. British Pregnancy Advisory Service* [1979] QB 276.

35 Here we use the term ‘woman’ despite its gendered limitations because this is the language deployed in the Abortion Act 1967.

36 S.1 (1) (a) Abortion Act 1967, as amended by s.37 Human Fertilisation and Embryology Act 1990. The further three grounds in the Act include where termination is necessary to prevent grave permanent injury to a woman’s physical or mental health; where termination presents a lesser risk to the woman’s life than continuing pregnancy; or where there is a substantial risk the fetus is handicapped; s.1 (1) (b)–(d).

37 Earlier in this paper, we discussed the variance in guidance on the gestational limit for safe EMA.

38 Hervey and Sheldon, *supra* note 3, at 126.

39 Elizabeth Chloe Romanis, Jordan Parsons and Nathan Hodson, *COVID-19 and Remote Access to Abortion Care: An Update*, https://blogs.bmj.com/bmjsrh/2020/03/23/covid-19-abortion-update/ (accessed Mar. 23, 2020).

40
*British Pregnancy Advisory Service v Secretary for State for Health* [2012] 1 W.L.R. 580.

41 Scottish Minister for Public Health, *The Abortion Act 1967 (Place for Treatment for the Termination of Pregnancy) (Approval) (Scotland)*, https://www.sehd.scot.nhs.uk/cmo/CMO(2017)14.pdf (accessed Feb. 7, 2020).

42 Welsh Minister for Health and Social Services, *The Abortion Act 1967 (Approval of Place for Treatment for the Termination of Pregnancy) (Wales)*, https://gov.wales/sites/default/files/publications/2019-07/the-abortion-act-1967-approval-of-place-for-treatment-for-the-termination-of-pregnancy-wales-2018-2018-no-56.pdf (accessed Feb. 7. 2020).

43 Secretary of State for Health and Social Care, *The Abortion Act 1967—Approval of a Class of Places*, https://assets.publishing.service.gov.uk/government/uploads/system/uploads/attachment_data/file/768059/Approval_of_home_use_for_the_second_stage_of_early_medical_abortion.pdf (accessed Feb. 7, 2020).

44 Per the Approval Orders issued by the relevant ministers and affirmed in *SPUC Pro-Life Scotland Limited v Scottish Ministers* (2018) S.L.T 1033 at para 30 per Lady Wise.

45 This order has since been removed from the government website but was summarized here: Romanis, Parsons and Hodson, *supra* note 39.

46 Elizabeth Chloe Romanis, Jordan Parsons & Nathan Hodson, *COVID-19 and Abortion Care Update: Department of Health and Social Care ‘Error’*  https://blogs.bmj.com/bmjsrh/2020/03/24/covid-19-abortion-3/ (accessed Mar. 24, 2020).

47 Denis Campbell, *GPs told to Switch to Digital Consultations to Combat Covid-19*,’ https://www.theguardian.com/world/2020/mar/06/gps-told-to-switch-to-remote-consultations-to-combat-covid-19 (accessed Mar. 24, 2020).

48 British Pregnancy Advisory Service, *Open Letter to: Rt Hon Matt Hancock MP, Secretary of State for Health*, https://drive.google.com/file/d/1TujbubXHjaN7H6FD2U5CvZvtFTmqjCXD/view (accessed Mar. 30, 2020).

49 Department of Health and Social Care, *The Abortion Act 1967—Approval of a Class of Places*. (The new approval order has yet to be posted online), para 4 (a). https://assets.publishing.service.gov.uk/government/uploads/system/uploads/attachment_data/file/876740/30032020_The_Abortion_Act_1967_-_Approval_of_a_Class_of_Places.pdf (accessed May 23, 2020).

50
*Id.*

51 Note that the Scottish approval order allows for remote access to EMA until 11 weeks 6 days' gestation; which is two weeks longer than it is lawful in England and Wales. Scottish Government, *The Abortion Act 1967 (Place for Treatment for the Termination of Pregnancy) (Approval) (Scotland) 2020*, https://www.sehd.scot.nhs.uk/cmo/CMO(2020)09.pdf (accessed Apr. 27, 2020).

52 Welsh Government, *The Abortion Act 1967—Approval of a Class of Place for Treatment for the Termination of Pregnancy (Wales) 2020*, https://gov.wales/sites/default/files/publications/2020-04/approval-of-a-class-of-place-for-treatment-for-the-termination-of-pregnancy-wales-2020.pdf (accessed Apr. 27, 2020).

53 Though it is not the case that both doctors must consult the patient as the Abortion Act 1967 contains no requirement that doctors forming the opinion that abortion is lawful to have consulted or examined the pregnant person accessing care: Department of Health, *Guidance in Relation to the Requirements of the Abortion Act 1967*, https://assets.publishing.service.gov.uk/government/uploads/system/uploads/attachment_data/file/313459/20140509_-_Abortion_Guidance_Document.pdf (accessed Apr. 29, 2020) at para 12.

54 Welsh Government, *supra* note 52.

55 Scottish Government Chief Medical Officer Directorate, *Abortion—Covid-19—Approval for Mifepristone To Be Taken At Home And Other Contingency Measures*, https://www.sehd.scot.nhs.uk/cmo/CMO(2020)09.pdf (accessed Apr. 28, 2020).

56 S.9 (2) Northern Ireland (Executive Formation etc.) Act 2019.

57 HM Government, *A New Framework for Abortion Services in Northern Ireland*, https://assets.publishing.service.gov.uk/government/uploads/system/uploads/attachment_data/file/875380/FINAL_Government_response_-_Northern_Ireland_abortion_framework.pdf (accessed Apr. 29, 2020).

58 The Abortion (Northern Ireland) Regulations 2020, regulation 8.

59 Elizabeth Romanis & Jordan Parsons, *Access to Remote Abortions Services should not be Temporary*, https://blogs.bmj.com/bmj/2020/04/02/elizabeth-chloe-romanis-jordan-parsons-access-remote-abortion-services-should-not-temporary/ (accessed Apr. 28, 2020); Sheelagh McGuiness & Jane Rooney, *A Legal Landmark in Reproductive Rights: The Abortion (Northern Ireland) Regulations 2020*, https://legalresearch.blogs.bris.ac.uk/2020/04/a-legal-landmark-in-reproductive-rights-the-abortion-northern-ireland-regulations-2020/ (accessed Apr. 28, 2020).

60 Alliance for Choice, *Home Abortion Care Now!*, https://mailchi.mp/60d335e9b6ca/stormont-delays-so-activists-step-in-for-telemedicine (accessed Apr. 29, 2020); British Pregnancy Advisory Service, *BPAS Launches Emergency Pills by Post for Women in Northern Ireland Amid Shameful Political Gameplay with Women’s Health during the Covid-19 Pandemic*, https://www.bpas.org/about-our-charity/press-office/press-releases/bpas-launches-emergency-abortion-pills-by-post-for-women-in-northern-ireland-amid-shameful-political-gameplay-with-women-s-health-during-the-covid-19-pandemic/ (accessed Apr. 29, 2020).

61
*Roe v Wade* US 113 (1973).

62
*Id.* at 160 per Blackmun J.

63
*Planned Parenthood v. Casey* 112 US 2791 (1992).

64
*Id*. at 860 per O’Connor J., Kennedy J. & Souter J.

65 Interestingly, the Supreme Court has failed to quantify a precise point of viability, so it is left to State legislators to determine; and it is thus defined slightly differently in different States.

66 Arizona expanded telemedicine laws in order to encourage remote access to healthcare in 2019 in SB 109 but expressly banned abortion being provided by telemedicine. Planned Parenthood Arizona is currently pursuing legal action in the ninth circuit *Planned Parenthood Arizona Incorporated et al. v. Brnovich et al.* 4:2019cv00207; these proceedings were instigated in 2019. There are also several provisions in the State Statutes that expressly state that a woman must be treated or consulted in person, for example, ARS §§ 36-2153(A) (1) and (2) (2019). In an earlier case challenging these provisions, *Planned Parenthood Arizona Incorporated v. Association of Pro-Life Obstetricians & Gynecologists* 227 Ariz. 262, 257 P.3d 181 (Ct. App 2011), the Court of Appeals of Arizona found that the challenge failed because there were sufficient grounds to believe that care provided by a physician in-person was more effective.

67 Arkansas Telemedicine Ban (HB 1076), passed in 2015, requires a woman and the physician be in the same room during the administration of abortion medication and notes that the limitations in the Bill ‘do not affect telemedicine practice that does not involve the use of mifepristone or another drug or chemical to induce an abortion.’ The law reads that ‘the initial administration of the drug or chemical [to induce abortion] shall occur in the same room and in the physical presence of the physician who prescribed, dispensed, or otherwise provided the drug or chemical to the patient:’ AR Code § 20-16-603 (2018). This is a direct ban on TEMA because telemedical services were mentioned in the bill amending the Arkansas Code. A challenge was brought against the prevention of TEMA in *Planned Parenthood of Arkansas v. Jegley* 864 F.3d 953 (8th Cir 2017) but failed on the grounds that the Planned Parenthood had failed to establish (by defining or estimating) the number of pregnant people who might be unduly burdened by an effective ban on TEMA.

68 Indiana state law requires that a woman be examined in person before being prescribed abortion services. The statute expressly says that ‘in person’ does not include the use of telehealth or telemedicine services.’ IN Code § 16-34–2-1 (2019).

69 The South Carolina Code explicitly prohibits the use of telemedicine in medical abortion; ‘prescribing abortion-inducing drugs is not permitted; as used in this article ‘abortion-inducing drug’ means a medicine, drug, or any other substance prescribed or dispensed with the intent of terminating the clinically diagnosable pregnancy of a woman:’ SC Code § 40-47-37 (2019).

70 West Virginia Bill Relating to the Practice of Telemedicine (HB 2509), passed in 2017, expressly states in its list of exemptions to telemedical prescriptions that ‘a physician or healthcare provider may not prescribe any drug with the intent of causing an abortion’: WV Code § 30-14-12d (2019).

71 Alabama state law places a requirement on physicians to physically examine patients before the prescription of abortion medications: AL Code § 26-23E-7 (2019).

72 Oklahoma requires that abortions ‘otherwise permitted by law must be performed only in a hospital, as defined in this article, which meets standards set by the [State] Department’ of Health 63 OK § 63-1-737 (2019).

73 Louisiana Telemedicine Ban (SB 90), passed in 2014, specifies that ‘the physician who prescribed the drug or chemical [for the procurement of abortion] shall be in the same room and in the physical presence of the pregnant woman when the drug or chemical is initially administered, dispensed, or otherwise provided to the pregnant woman:’ LA Rev Stat § 40:1061.11 (2018).

74 The Mississippi Code specifies that ‘the physician giving, selling, dispensing, administering or otherwise providing or prescribing the abortion-inducing drug must first physically examine the woman:’ MS Code § 41-41-107 (2018).

75 Missouri Telemedicine Ban (HB 400), passed in 2013, introduced a requirement that ‘when RU-486 (mifepristone) or any drug or chemical is used for the purpose of inducing an abortion, the initial dose of the drug or chemical shall be administered in the same room and in the physical presence of the physician who prescribed, dispensed, or otherwise provided the drug or chemical to the patient:’ MO Rev Stat § 188.021 (2019).

76 The abortion statute Neb. Rev. Stat. Ann § 28-335 (2019) specifically requires that the licensed physician performing an abortion be ‘physically present in the same room with the patient when’ performing the abortion.

77 Abortion can only be lawfully provided in North Carolina ‘in a hospital or clinic certified by the Department of Health and Human Services to be a suitable facility for the performance of abortions:’ NC Gen Stat § 14-45.1 (2019).

78 The Abortion Control Act in North Dakota specifies that ‘when an abortion-inducing drug or chemical is used for the purpose of inducing an abortion, the drug or chemical is used for the purpose of inducing an abortion, the drug or chemical must be administered by or in the same room and in the physical presence of the physician who prescribed, dispensed or otherwise provided the drug or chemical to the patient:’ ND Cent. Code Ann. § 14-02.1-03.5 (2019).

79 In South Dakota the law specifically requires that ‘no surgical or medical abortion may be scheduled except by a licensed physician and only after the physician physically and personally meets with the pregnant mother, consults with her, and performs an assessment of her medical and personal circumstances:’ SD Codified L § 34-23A-56 (2019).

80 The Tennessee Code specifies that no licensed physician ‘shall perform or attempt to perform any abortion, including a medically induced abortion, or shall prescribe any drug or device intended to cause a medical abortion, except in the physical presence of the pregnant woman. No drug or device intended to cause a medical abortion shall be administered or dispensed to a pregnant woman except in the physical presence of her physician:’ TN Code § 63-6-241 (2018).

81 Texas Omnibus Abortion Bill (HB 2), passed in 2013, contained a significant number of restrictions on abortion provision—including a provision that a physician dispensing medication for abortion must ‘examine the pregnant woman,’ implying that there must be a face-to-face consultation. Tex. Health & Safety Code § 171.063 (2017).

82 Wisconsin law mandates that a physician be ‘physically present in the room when the drug is given to the woman:’ WI Stat § 253.105 (2019). The ‘physician only’ requirement means that a physician—and only a physician—must oversee a woman being given abortion medications and therefore precluding telemedical services. Legal action has been instigated against this law (and other aspects of the Wisconsin statutes) in the US District Court Western District of Wisconsin; *Planned Parenthood of Wisconsin Incorporated et al. v. Joshua Kaul et al.* case no. 19-cv-38.

83
*Planned Parenthood of the Heartland Incorporated v. Iowa Board of Medicine* 865 N.W.2D 252 (2015) at 254 per Justice Wiggins. Note that this law in Iowa has been permanently enjoined as a result of this decision.

84
*supra* note, at 80.

85 Office of the Texas Governor, *Executive Order by the Governor of Texas*, https://gov.texas.gov/uploads/files/press/EO-GA_09_COVID-19_hospital_capacity_IMAGE_03-22-2020.pdf (accessed Mar. 26, 2020).

86 Texas Attorney General, *supra* note 11.

87 Office of the Texas Governor, *Governor Abbott Issues Executive Order to Loosen Restrictions on Surgeries*, https://gov.texas.gov/news/post/governor-abbott-issues-executive-order-to-loosen-restrictions-on-surgeries (accessed Apr. 29, 2020).

88
*supra* note 81.

89 State of Mississippi Office of the Governor, *Executive Order No. 1463*, https://www.msema.org/wp-content/uploads/2020/03/Executive-Order-1463.pdf (accessed Mar. 26, 2020).

90 Ohio Telemedicine Medication Abortion Ban (SB 260) was passed in the senate on March 4, 2020, and has now been passed to the house for hearings. Ohio currently has very restrictive abortion law regardless: Ohio Rev Code § 2919.12 (2019) that is likely to constitute hurdles to TEMA in themselves.

91 Ohio Department of Health, *supra* note, at 11.

92 Hannah Knowles, *Ohio Clinics Ordered to Halt Abortions Deemed ‘Nonessential’ Amid Coronavirus Response*, https://www.washingtonpost.com/health/2020/03/21/ohio-abortion-clinics-coronavirus/ (accessed Mar. 26, 2020); it is important to note that clinics in Ohio remain open and are compliant with guidance about safe healthcare provision at this time.

93 Kellie Copeland in Allan Smith, *Texas, Ohio, Order Clinics to Halt Abortion Procedures Amid Coronavirus*, https://www.nbcnews.com/politics/politics-news/texas-ohio-order-clinics-halt-abortion-procedures-amid-coronavirus-n1167201 (accessed Mar. 26, 2020); It is to be noted that in other states, such as Massachusetts and Washington, officials have taken the opposite approach and affirmed that abortion services are not ‘nonessential services’ for the purposes of measures limiting healthcare during the pandemic.

94 See our above figures on the number of people we estimate need or will need abortions during the next few months.

95 Meaning the 12 states participating in the TelAbortion Project.

96
*New State Ice Co. v. Liebmann*. 285 U.S. 262 at 56.

97 British Pregnancy Advisory Service, *Pills by Post—Remote Abortion Pill Treatment*, https://www.bpas.org/abortion-care/abortion-treatments/the-abortion-pill/remote-treatment/ (accessed Apr. 28, 2020).

98 That is not to say that the process of TEMA being permitted throughout the US would be simple, rather the practical element would be easily mirrored if permitted. Further obstacles will inevitably exist given the extent of current legal barriers in the US.

99 Yaneer Bar-Yam Y., *We Need an Immediate Five-Week National Lockdown to Defeat Coronavirus in America*, https://eu.usatoday.com/story/opinion/2020/03/21/coronavirus-america-needs-five-week-national-lockdown-column/2890376001/ (accessed Mar. 23, 2020); New York Post, *How Long will New York’s Coronavirus Lockdown Last?*, https://nypost.com/2020/03/20/how-long-will-new-yorks-coronavirus-lockdown-last/ (accessed Mar. 20, 2020).

100 See discussion of risk threshold above.

101 Nathan Hodson, Elizabeth Chloe Romanis & Jordan Parsons, *Abortion and Covid-19: MPs should Beware Anti-Abortion Letters*, https://blogs.bmj.com/bmjsrh/2020/03/26/abortion-covid19-letters/ (accessed Mar. 26, 2020).

102 Casey, *supra* note 63, 860 per O’Connor J., Kennedy J. & Souter J.

103 Lisa M. Kelly, *Abortion Travel and the Limits of Choice*, 12 FIU L. Rev. 27 (2016).

104 We have limited the scope of our inquiry to the COVID-19 disaster. However, the authors are of the opinion that access to TEMA is also necessary even in the absence of a pandemic.

105 As noted earlier in this paper, both England and Wales have dated sunset clauses, whereas Scotland’s order merely states that they intend to reverse the change following the pandemic.

106
*A, B and C v. Ireland* [2010] ECHR 2032 at para 7 per Judge Geoghegan.

107
*Id.*

108 Rosamund Scott, *Risks, Reasons and Rights: The European Convention on Human Rights and English Abortion Law*, 24 Med. LR 1 (2016), at 4–5.

109
*Brüggemann and Scheuten v. Federal Republic of Germany* (1981) 3 EHRR 244, para 59.

110 A, B and C v. Ireland, *supra* note 106; Tysiąc v. Poland [2007] ECHR 212; RR v. Poland [2011] ECHR 828.

111 Though, she notes that the boundary between positive and negative obligations is not always clear. Scott, *supra* note 108, at 7.

112 This matter is settled in Great Britain; people do not have a right to access abortion under the law, but abortion can be lawfully facilitated by doctors. There are reasons to challenge the framing of the Abortion Act for this reason, but they are beyond the scope of this paper.

113 The right to private life is to be interpreted broadly as a right to live a personal life as one sees fit without state interference. It also encompasses a right to physical integrity.

114 Scott, *supra* note 108, explains that this is the key test regarding the positive obligations—we are applying this in context.

115 Tysiąc v. Poland, *supra* note 110, at 116 [emphasis added].

116 Scott, *supra* note 108, at 19 [emphasis in original].

117 RR v. Poland, *supra* note 110.

118
*Id.* at 199.

119 Scott notes that the two-signature requirement in the AA 1967 is itself something that is in need of reconsideration because of the UK’s positive obligations under Article 8 resulting from the reduced margin of appreciation after having made legal provision for abortion. Scott, supra note 108, at 23.

120 As per the requirements of S.1 (1) (a) Abortion Act 1967.

121 Article 8 (2) of the European Convention on Human Rights specifies that ‘there shall be no interference by a public authority with the exercise of this right except as in accordance with the law and is necessary in a democratic society in the interests of national security, public safety or the economic well-being of the country, for the prevention of disorder or crime, for the protection of health or morals, or for the protection of the rights and freedoms of others.’

122 Lawless v. Ireland (no. 3) (1961) 1 EHRR 15.

123 Article 15 European Convention on Human Rights.

124 As per Article 15 (3) European Convention on Human Rights.

125 Ireland v. United Kingdom [1978] ECHR 1.

126
*Id.*

127
*Id.*

128 Kudla v. Poland [2000] ECHR 512; Paladi v. Moldova [2008] 47 EHRR 15; Amirov v. Russia [2014] ECHR 1330.

129 Kudla v. Poland *supra* note 128.

130
*Id.* at 91.

131 A, B and C v. Ireland, *supra* note 106.

132
*Id.* at 162.

133
*Id.* at 163.

134
*Id.* at 164.

135 Tysiąc v. Poland, *supra* note 110, at 65.

136
*Id*.

137
*Id.* at 66.

138 RR v. Poland, *supra* note 110, at 153–162.

139
*Id.* at 159.

140
*Id.*

141
*Id.*

142
*Id.* at 160.

143
*Id.* at 159.

144
*Id.* at 155.

145 Physical symptoms of early pregnancy include morning sickness, lethargy, sore breasts, urgency, constipation, and strange tastes and smells. National Health Service, *Signs and Symptoms of Pregnancy*, https://www.nhs.uk/conditions/pregnancy-and-baby/signs-and-symptoms-pregnancy/ (accessed Mar. 30, 2020).

146 Studies have found that there are significant adverse psychological impacts in experiencing unwanted pregnancy. M. Antonia Biggs et al., *Women’s Mental Health and Well-being 5 Years After Receiving or Being Denied an Abortion*, 74 JAMA Psychiatry 169 (2017), at 174.

147
*Id.* at 174.

148 Several national newspapers misreported the legalities of TEMA in Wales and Scotland. See: Romanis and Parsons, *supra* note 59.

149 Article 15 (2) European Convention on Human Rights.

150
*Casey, supra* note 63, at 878 per O’Connor J., Kennedy J. & Souter J.

151 For example, in Casey, *supra* note 63, it was found that a mandatory 24-hour waiting period was not an undue burden.

152 Whole Women’s Health et al. v. John Hellerstedt et al., 579 U.S. (2016).

153
*Id.* at 19–20 per Breyer J.; This is a reiteration of the point established in Casey that when considering whether a law constitutes an undue burden, Casey, *supra* note 63, instructs that the burdens that a legislative provision places on access to abortion alongside any potential benefits that the law might confer: at 887–898 per O’Connor J., Kennedy J. and Souter J.

154
*Id.* at 19 per Breyer J.

155 Kate L. Fetrow, *Taking Abortion Rights Seriously: Toward a Holistic Undue Burden Jurisprudence*, 70 Stan. L. Rev. 319 (2018), 324.

156 Gonzales v. Carhart 550 U.S. 124 (2007).

157
*Id.* at 165; Hellerstedt*, supra* note 152, at 20–21.

158 Planned Parenthood Arizona Incorporated v. Association of Pro-Life Obstetricians & Gynecologists, *supra* note 74.

159 Planned Parenthood of Arkansas v. Jegley, *supra* note 67.

160 Endler and others, *supra* note 29.

161 Hellerstedt*, supra* note 152, per Breyer J.

162 Justice Breyer considered evidence related to complication rates and found the requirement for admitting privileges wanting, on the basis that the medical evidence conclusively showed that there were low complication rates for abortion. Therefore, it could not be said that this burden that placed a significant obstacle on clinics providing abortion access helped meet the objective of protecting pregnant people’s health. Therefore, the requirement was unconstitutional.

163 Roe v. Wade, *supra* note 61.

164 See for example, Arizona, *supra* note 66.

165 Hellerstedt*,* supra note 152, at 30 per Breyer J.

166 Planned Parenthood of the Heartland Incorporated v Iowa Board of Medicine, *supra* note 83, at 269 per Justice Wiggins.

167
*Id.* at 263–264 per Justice Wiggins.

168 We note here that there are other medical practitioners qualified, even if not legally enabled in GB and the US, to provide abortion care.

169 In re: Gregg Abbott, No. 20-50264 (5^th^ Cir. 2020) [emphasis in original].

170 Elisabeth Smith at the Sexual and Reproductive Health Journal Webinar, *COVID-19: What Implications for Sexual and Reproductive Health and Rights Globally?* (accessed Mar. 27, 2020).

171 Planned Parenthood v. Abbott, https://reproductiverights.org/sites/default/files/2020-04/19A-xxxx%20-%20Planned%20Parenthood%20Emergency%20Application%20to%20Justice%20Alito%20-%20FINAL_%28179588103%29_%284%29.pdf (accessed Apr. 29, 2020).

172 Jessica Mason Pieklo, *How COVID-19 Could Bring the End of Roe v. Wade*, https://rewire.news/article/2020/04/03/covid-19-could-bring-the-end-of-roe-v-wade/ (accessed Apr. 29, 2020).

173 Office of the Texas Governor, *supra* note 87.

174 Ramos v. Louisiana, 590 U.S. (2020).

175
*Id.*

176 We are grateful to an anonymous reviewer for this point.

